# A letter to the editors discussing the following article: “Complicated COVID-19 in pregnancy, maternal and neonatal outcomes: a case report” by MOUNA GARA *et al*.

**DOI:** 10.11604/pamj.2022.42.81.34919

**Published:** 2022-05-31

**Authors:** Soukaina Abidi, Youssra Azzouz, Saliha Chbicheb

**Affiliations:** 1 International University of Rabat, College of Health Sciences, International Faculty of Dental Medicine, Biomed Unit, Rabat, Morocco,; 2 Department of Oral Surgery, Faculty of Dentistry, Mohamed V University, Rabat, Morocco

**Keywords:** Pregnancy, COVID-19, vaccine

## To the editors of the Pan African Medical Journal

I'm an avid reader of The Pan African Medical Journal and It´s with a great interest that I have read this following article: “Complicated COVID-19 in pregnancy, maternal and neonatal outcomes: a case report” [1]. Physiological, mechanical, and immunologic alterations in pregnancy could potentially affect the susceptibility to and the severity of COVID-19 during pregnancy [2]. Owing to the lack of comparable incidence data and the challenges with disentangling differences in the susceptibility from different exposure risks, the data are insufficient to determine whether pregnancy increases the susceptibility to SARS-CoV-2 infection. Evidence is accumulating that SARS-CoV-2 infection during pregnancy is associated with a number of adverse pregnancy outcomes including preeclampsia, preterm birth, and stillbirth, especially among pregnant persons with severe COVID-19 disease [2-3].

While several treatments such as Remdesivir and Dexamethasone are either available or in development for severe COVID-19 [3], early testing and treatment remains the key for a better prognosis. The patient described in this case report waited about a week of showing COVID-19 symptoms. COVID-19 should be treated early and hard: this approach has been proven to be successful in thousands of patients. The earlier you treat, the better the outcomes. If you wait five days after experiencing symptoms before being treated, your chance of being hospitalized can rise from zero to 10% or more [4]. The only hospitalizations we´ve seen in practice are when the patient waits too long before seeing his or her doctor, can´t get a prescription filled in a timely manner, doesn´t take the drug, or the drug quality is an issue. The first two are the most common. The later you start treatment, the longer it may take you to recover and your outcomes will be more unpredictable. If you wait more than 48 hours beyond the onset of first symptoms, drug treatment will take longer to have a positive effect, higher doses and more drugs will be needed, and your outcomes will be more unpredictable [3-5].

In 2022 and two years after its release, the COVID-19 vaccine is considered to be the number one tool to bring this pandemic to an end. A large share of the world needs to be immune to the virus. Vaccines are a technology that humanity has often relied on in the past to bring down the death toll of infectious diseases [6]. The vaccinal status of the patient described in this article hasn´t been revealed. This leads us to think that the patient is not fully nor partially vaccinated. An ounce of prevention is worth a pound of cure. Safe and effective vaccines are a game-changing tool: but for the foreseeable future we must continue wearing masks, cleaning our hands, ensuring good ventilation indoors, physically distancing and avoiding crowds. Although the data are sparse, they are so far reassuring. For this reason, regulatory bodies in the United Kingdom, European Union and United States have recommended that pregnant people should be offered the vaccine where the benefits outweigh the potential risks: pregnant workers on the frontline and those with pre-existing conditions are now receiving the vaccine [6]. The U.S. Food and Drug Administration (FDA) issued an Emergency Use Authorization (EUA) for the following vaccines along with the Indian Central Drugs and Standards Committee (CDSCO) in the type of COVID-19 vaccines authorized for emergency use for pregnant women): Pfizer-BioNTech - Moderna - Janssen Biotech, Inc. (Johnson & Johnson)- Serum Institute of India (Covishield) - Bharat Biotech (Covaxin) [7].

While during pregnancy women's immunological, cardiovascular, and respiratory systems undergo significant physiological changes, perhaps a little amount of stress can exacerbate the disease severity. Pregnancy, on the other hand, produces hypercoagulability, endangering lives due to venous and arterial thromboembolism. According to early evidence and observational research, COVID-19 patients have a higher risk of thrombosis. Respiratory viral infections during pregnancy have been linked to factors such as low birth weight and preterm birth in several studies [6]. Additionally, high fever in early pregnancy may increase the chances of certain birth defects. In terms of vaccine effectiveness, studies indicate that the administration of the mRNA vaccines results in a robust maternal humoral response. Although the antibody response to vaccination among pregnant persons has not been rigorously compared with the response among nonpregnant persons, there is no reason to expect differences [7]. Furthermore, maternal immunoglobulin G antibodies efficiently cross the placenta, resulting in relatively high titers in the fetus [6-7].

## Conclusion

The weeklong period of time the patient has waited before hospital admission, especially on a high-risk pregnancy because of the weight gain and obesity (BMI 36 kg/m^2^) might have been crucial in the development of the symptoms and complications [8]. The local authorities have to put the emphasis on the value of vaccination among high risk individuals especially pregnant women.
